# Is human albumin injection the best choice to treat hypoalbuminemia?

**DOI:** 10.1371/journal.pone.0311949

**Published:** 2024-10-21

**Authors:** Yingqin Shi, Hai Song, Lingzhi Fang, Fuxi Wu, Bo Qiu, Dongdong Tian, Caixia Liu, Hailing Di

**Affiliations:** 1 Department of Pharmacy, Hebei General Hospital, Shijiazhuang, China; 2 Department of Science and Education, Tangshan People’s Hospital, Tangshan, China; 3 The sales department, Fresenius Kabi Huarui Pharmaceutical Co., Ltd, Beijing, China; 4 Clinical Nutrition Department, Hebei Medical University Third Hospital, Shijiazhuang, China; Delta State University, NIGERIA

## Abstract

**Background:**

Currently, there is an irrational use of drugs in the treatment of hypoproteinemia. This study evaluates the value of 12 injections to provide a basis for drug selection for the treatment of hypoproteinemia.

**Methods:**

The content of the first restrictive amino acid, the comprehensive quality of the total essential amino acid, and the closeness to the whole egg protein or FAO/WHO model were evaluated to compare their value in the amino acid synthesis of human serum albumin. A comparison was made between the matching degree of HA and therapeutic amino acids with the human plasma amino acid profile. Furthermore, the value and safety of synthetic human serum albumin were compared.

**Results:**

The lowest synthetic value of human serum albumin was 18AA-V, and the highest was 18AA-II. The CS values of HA, 18AA-V, 18AA-II, 18AA-Ip, 18AA-II_p_, and 19AA-I_p_ were 0.18, 0.58, 0.78, 0.55, 0.54, and 0.59, respectively. The similarity to egg protein was 0.81, 0.92, 0.94, 1.00, 1.18, and 1.18, respectively. The proximity values to FAO/WHO standards were 0.81, 0.85, 0.90, 1.36, 1.54, and 1.54, respectively. The changes in 3AA and the amino acid profile were matched when liver function was abnormal. When the renal function was abnormal, 9AA was matched. During trauma, 18AA-VII was matched. The amino acid profile of HA did not correspond.

**Conclusion:**

Patients with normal liver and kidney function should choose compound balanced amino acid injection, while patients with abnormalities should choose 3AA. Patients with renal dysfunction should choose 9AA. Trauma patients should choose 18AA-VII.

## 1. Introduction

Human albumin (HA) injection is a blood product separated and purified from healthy human plasma. The recognized indications for HA include shock caused by post-traumatic bleeding or burns, post-traumatic brain edema and elevated intracranial pressure, edema or ascites caused by cirrhosis or kidney disease, neonatal hyperbilirubinemia, adjuvant therapy for burns and extracorporeal circulation, prevention and treatment of adult respiratory distress syndrome, and hypoalbuminemia. Due to the unique source of HA, the clinical dosage is on the rise, and the market shortage is becoming increasingly pronounced [1]. It has been reported that the proportion of patients in the United States who receive an irrational rate of HA is 57.8%. Furthermore, albumin therapy for serum albumin values of less than 2 g/dL is inappropriate in 100% of cases [2]. There are research reports in Iran that the unreasonable use rate of hyaluronic acid exceeds 70%. Hypoalbuminemia is one of the most common reasons for unreasonable prescription of albumin. Nutritional support is another reason for the unreasonable prescription of albumin [3]. A study in China showed that out of 1984 patients, 1044 (52.6%) had improper use of HA. The main indications for irrational drug use are hypoalbuminemia (30.0%) and nutritional support (21.9%) [4]. There are many main causes of hypoalbuminemia such as malnutrition or malabsorption. When liver function is impaired, the liver’s ability to synthesize albumin decreases, and long-term protein loss is excessive (such as gastrointestinal ulcers, hemorrhoids, excessive menstruation, nephrotic syndrome, etc.). In addition, there are reasons such as accelerated protein breakdown, rapid consumption (severe infections, malignant tumors, etc.), and trauma [5]. Any cause of hypoalbuminemia requires treatment. Amino acids are the basic substance of protein [6]. At present, there are various types of amino acid injections available on the domestic market. For example, adult compound balanced amino acid injections (CBAAI), pediatric CBAAI, and therapeutic amino acids developed based on liver and kidney function and different diseases of patients. The latter include compound amino acid injection 3AA for liver disease, compound amino acid injection 9AA for kidney disease, and compound amino acid injection 18AA-VII for trauma. Regardless of the type of amino acid, its function is to synthesize HA and provide nutritional support, and it is inexpensive and easy to obtain. This study evaluates the value of synthesizing HA using 5 types of adult balanced amino acid injections (18AA, 18AA-I, 18AA-II, 18AA-IV, 18AA-V), 3 types of pediatric CBAAI (18AA-I_p_, 18AA-II_p_,19AA-I_p_), and 3 types of therapeutic compound amino acid injections (3AA, 9AA, 18AA-VII) that have been marketed in China. It aims to provide a basis for the selection of drugs for treating hypoalbuminemia and a reference for reducing the unreasonable use of HA.

## 2. Materials

### 2.1 Data sources

The total number of amino acid residues and the proportion of various amino acids in the HA structure are derived from literature [7]. The molecule of HA consists of a single polypeptide chain and contains 585 amino acid residues: Lys_59_, His_16_, Arg_24_, Asp_53_, Thr_28_, Ser_24_, Glu_82_, Pro_24_, Gly_12_, Ala_62_, Cys_35_, Val_41_, Met_6_, Ile_8_, Leu_61,_ Tyr_18_, Phe_31_, and Trp. According to the amino acid composition, the molecular weight of HA is calculated to be 66,500.

The prescription composition of 5 varieties of adult CBAAI comes from literature [8]. The prescription composition of paediatric CBAAI and therapeutic compound amino acid injections comes from the drug instructions (Table 1).

**Table 1:** The prescription composition of paediatric CBAAI and therapeutic compound amino acid injections.

	18AA-I_p_	18AA-II_p_	19AA-I_p_	3AA	9AA	18AA-VII
Lysine	7.90	6.9	4.9		9	2
Valine	3.60	4.7	4.7	1.05	6.4	2.8
Threonine	3.60	2.5	2.5		4	1.5
Isoleucine	3.10	4.9	4.9	13.5	5.6	1.82
Tryptophan	1.40	1.2	1.2		2	0.26
Methionine + cysteine	1.30+1.00	2.0+0.20	2.0+0.20		8.8+0.10	0.88+0.07
Leucine	7.00	8.4	8.40	16.5	8.8	2.58
Phenylalanine + Tyrosine	2.7+0.6	2.9+1.4	2.9+1.4		8.8	1.40+0.08
Sigma EAA	32.20	34.25	32.25			
Histidine	2.1	2.9	2.9		2.5	1
Proline	5.6	4.1	4.1			1
Serine	3.8	2.3	2.3			0.34
Glutamic acid	7.1	3	3			0.1
Alanine	6.3	3.2	3.2			1.42
Arginine	4.1	7.3	7.3			1.8
Aspartic acid	4.1	1.9	1.9			0.2
Glycine	2.1	2.2	2.2			1.4
Taurine	0	0.15	0.15			
Sigma AA	61.4	55.65	53.65			

## 3. Methods

### 3.1 Evaluation on the value of HA and CBAAI in synthesizing HA

#### 3.1.1 Evaluation of the first limiting amino acid

The relative content of one or several essential amino acids (EAA) in a protein or CBAAI is relatively low. The amino acid with the lowest content is called the first limiting amino acid. The lower the content of the first restricted amino acid, the less it can synthesize human protein, and the lower the value of its synthesis of HA.

Taking egg protein as the standard mode for chemical score (CS) [9]: CS is employed to assess the proximity of the relative content of a specific EAA in the total EAA in protein or CBAAI to that of the corresponding EAA in the total EAA in standard egg protein. The closer the CS value to 1, the higher its value of synthesis of HA.

$$CS=aa/AA_{EGG}$$ (1)

*aa* is the percentage of a specific amino acid in the test sample. *AA*_*EGG*_ is the percentage of the same amino acid in the whole egg protein.

Taking FAO/WHO as the standard mode for amino acid score (AAS) [10,11]: AAS is the ratio of the relative content of a certain EAA in protein or compound balanced amino acid injection to the total EAA and the corresponding amino acid content in the FAO/WHO scoring model. The closer the AAS value is to 1, the higher its value of synthesis of HA.


$$AAS=aa/AA_{FAO/WHO}$$ (2)

*AA*_*FAO/WHO*_ is the percentage of the amino acid in the FAO/WHO scoring standard mode?

#### 3.1.2 Evaluation of the comprehensive quality of EAA

For evaluating the quality of a specific protein or CBAAI, in addition to a certain EAA content, the comprehensive quality of the total EAA should be considered.

Taking egg protein as the standard mode for essential amino acid index (EAAI) [12]: EAAI is used for evaluating the comprehensive quality of a protein or CBAAI by considering the ratio of all EAA in the target protein to all EAA in egg protein. The closer the EAAI value is to 100, the higher its value of synthesis of HA.

$$EAAI={\left(100A/A_{E}*100/B*B_{E}*100C/C_{E}\cdots \right)}^{1/n}$$ (3)

n is the number of EAA. A, B, and C are the percentages of the EAA content of the detected amino acids. A_E_, B_E_, and C_E_ are the percentage of the EAA content of the whole egg protein.

Taking FAO/WHO as the standard mode for the score of the ratio coefficient of amino acid (SRCAA) [13]: Using amino acid balance theory and FAO/WHO EAA model, the ratio of amino acid (RAA) and ratio coefficient of amino acid (RCAA) of EAA in the evaluated protein and CBAAI are calculated. Then, SRCAA is calculated. The closer the SRCAA is to 100, the better synthesizes human serum protein, indicating a higher value of synthesis of HA.


$$\begin{array}{l} RAA=EAA\;content\;of\;the\;target\;protein\;(mg/g\;protein)\\ \;/corresponding\;EAA\;content\;in\;the\;WHO/FAO\;formula\;(mg/g\;protein) \end{array}$$ (4)

RCAA=averageofRAA/RAA (5)

SRCAA=100−CV×100 (6)

In the formula: CV is the coefficient of variation of RCAA. CV = standard deviation/mean.

Evaluation of the closeness of albumin total EAA to standard protein (whole egg protein or FAO/WHO model): According to the distance method of Lan and Apos, the closeness *μ*(*a*,*u*_*i*_) between subject *u*_*i*_ and standard protein a (whole egg protein or FAO/WHO model) is evaluated [14].


$$\mu (a,u_{i})=1-0.09\sum_{k=1}^{\infty }\frac{\left|a_{k}-u_{ik}\right|}{a_{k}+u_{ik}}$$ (7)

*a*_*k*_(*k* = 1,2,⋯,8) is the *k*^*th*^ EAA content (mg·g^-1^) of standard protein a (whole egg protein or FAO/WHO model). *u*_*ik*_(*k* = 1,2,⋯8) is the *i*^*th*^ content (mg·g^-1^) of each evaluated protein *k*^*th*^ EAA (K-type EAA corresponding to the standard protein).

The closeness value reflects the closeness of the protein quality of the evaluated protein and CBAAI to the standard protein (whole egg protein or FAO/WHO model). The closer the value is to 1, the better the synthesis of human serum protein and the higher its value of synthesis of HA.

### 3.2 Evaluation on the value of HA and therapeutic amino acid injections in synthesizing HA

Aromatic amino acids (AAA) in human body are metabolized by liver. Branched chain amino acids (BCAA) are metabolized by skeletal muscle. When liver function is impaired, AAA metabolism is slow. Its content increases in the body, while the content of BCAA decreases. This leads to a decrease in the molar concentration ratio of BCAA to AAA, which is directly proportional to the degree of liver injury. Drugs with high AAA content can cause abnormalities in brain tissue chemical neurotransmitters. BCAA can competitively inhibit AAA from penetrating the blood-brain barrier and participating in the metabolism of protein and sugar in the brain to prevent hepatic encephalopathy. Therefore, when treating hypoproteinemia in patients with abnormal liver function, drugs with high BCAA content and low AAA content should be selected [15]. Amino acid profiles of HA and compound balanced amino acid 3AA injection are compared to evaluate their value as a synthetic HA.

In patients with acute and chronic renal insufficiency and severe renal failure, the amino acid profile in the body has changed. The metabolism of EAA and semi EAA histidine has accelerated, and their content in the body has decreased. However, the metabolism of non-essential amino acid (NEAA) has slowed down, and their content in the body has increased. Therefore, when treating hypoalbuminemia in patients with abnormal renal function, drugs with high content of EAA and low content of NEAA should be selected to prevent azotemia and further damage to the kidney [15]. The value of HA synthesis is evaluated by comparing the amino acid profiles of HA and 9AA compound amino acid injections.

In trauma patients, amino acids decomposed from skeletal muscle proteins are the main source of amino acids in the body, especially the consumption of alanine, glutamine, and BCAA in the body. After trauma, the level of BCAA in the body decreases, causing amino acid imbalance and nutritional disorder. Therefore, exogenous supplement of BCAA has become an important means to correct negative nitrogen balance and promote skeletal muscle protein synthesis. When treating hypoalbuminemia in trauma patients, exogenous supplementation of high branched chain amino acids is necessary. This can greatly improve the composition of the patient’s plasma amino acid profile, promote protein synthesis, and inhibit protein breakdown [15]. The value of HA and compound amino acid 18AA-VII injection in synthesizing HA is evaluated by comparing their amino acid profiles.

### 3.3 Statistical methods

To ensure the validity of the study results, the value of HA and different amino acid injections in the synthesis of HA was statistically analyzed. The t test of two independent samples was used to compare the difference between HA and each amino acid injection. SPSS18.0 was used as statistical analysis software. The *P*<0.05 was used as the criterion for determining statistically significant differences.

## 4. Results

### 4.1 Evaluation results of the value of HA and CBAAI in the synthesis of HA

The molecular weight of HA molecule calculated from its amino acid composition was 66500^7^. The composition of EAA in HA molecule and EAA of HA were assessed (Table 2). Cysteine was synthesized from methionine. Tyrosine was structurally analogous to phenylalanine, and phenylalanine was biosynthesized from tyrosine within the human body. Therefore, cysteine and methionine were combined to calculate the former, and phenylalanine and tyrosine were combined to calculate the latter.

**Table 2:** Composition of EAA in HA molecule and mass ratio of EAA/HA.

	Molar mass of EAA(g/mol)	Number of EAA contained in HA	Molar mass of EAA * number of EAA in HA molecule (g/mol)	EAA of HA (mg/g)
Leucine	131.17	61	8001370	120.32
L-Isoleucine	131.17	8	1049360	15.78
Valine	117.15	41	4803150	72.23
Phenylalanine	165.19	31	5120890	77.01
Tyrosine	181.19	18	3261420	49.04
Tryptophan	204.22	1	204220	3.07
Methionine	149.21	6	8952600	13.46
Threonine	119.12	28	3335360	50.16
Lysine	146.19	59	8625210	129.7
Cysteine	121.16	35	424060	64.77
Methionine + cysteine			5135860	77.23
Phenylalanine + tyrosine			8382310	126.05

For the convenience of unified evaluation, the content of each EAA prescription g·1000mL^-1^ of three kinds of paediatric CBAAI was converted into the content of each EAA in the total amino acidmg·g protein^-1^ (Table 3).

**Table 3:** EAA of 18AA-I_p_, 18AA-Ⅱ_p_ and 19AA-I_p_.

	Whole egg protein	18AA-I	18AA-Ⅱ	19AA-I	FAO/WHO
Lysine	70.00	117.21	112.84	82.84	55.00
Valine	66.00	53.41	76.86	79.46	50.00
Threonine	47.00	53.41	40.88	42.27	40.00
Isoleucine	54.00	45.99	80.13	82.84	40.00
Tryptophan	17.00	20.77	19.62	20.29	10.00
Methionine + cysteine	57.00	34.12	35.98	37.19	35.00
Leucine	86.00	103.86	137.37	142.01	70.00
Phenylalanine + tyrosine	93.00	48.96	56.42	58.33	60.00
Sigma EAA	496.00	477.75	560.10	545.22	360.00

The CS and ASS of HA evaluation results are shown in Table 4.

**Table 4:** The CS and ASS evaluation results of HA.

	Whole egg protein	CS	HA	AAS	FAO/WHO
Lysine	70.00	1.53	129.70	1.43	55.00
Valine	66.00	0.90	72.23	0.87	50.00
Threonine	47.00	0.88	50.16	0.76	40.00
Isoleucine	54.00	0.24	15.78	0.24	40.00
Tryptophan	17.00	0.15	3.07	0.19	10.00
Methionine + Cysteine	57.00	1.12	77.23	1.34	35.00
Leucine	86.00	1.15	120.32	1.04	70.00
Phenylalanine + Tyrosine	93.00	1.12	126.05	1.27	60.00

The CS and ASS evaluation results of 18AA-II_original research_ and five formulations of amino acids for injection are shown in Table 5.

**Table 5:** The CS and ASS evaluation results of 18AA-II_original research_ and five formulations of amino acids for injection.

	18AA		18AA-I		18AA-II_original research_		18AA- II (8.5%)		18AA-IV		18AA-V	
	CS	AAS	CS	AAS	CS	AAS	CS	AAS	CS	AAS	CS	AAS
Lysine	1.09	1.02	1.13	1.05	1.62	1.52	1.62	1.52	1.50	1.40	1.37	1.28
Valine	0.97	0.94	1.05	1.02	1.00	0.97	1.00	0.97	0.58	0.56	0.59	0.58
Threonine	0.95	0.82	1.03	0.89	1.07	0.92	1.07	0.92	1.18	1.02	1.21	1.04
Isoleucine	1.16	1.15	1.16	1.15	0.93	0.92	0.93	0.92	0.88	0.88	0.91	0.90
Tryptophan	0.94	1.18	0.95	1.18	0.99	1.23	0.99	1.23	0.78	0.98	0.66	0.83
Methionine + Cysteine	0.70	0.84	0.58	0.69	0.88	1.05	0.88	0.88	0.74	0.88	0.76	0.91
Leucine	1.01	0.91	0.99	0.90	0.82	0.74	0.82	0.82	1.23	1.11	1.27	1.15
Phenylalanine + Tyrosine	1.07	1.22	1.04	1.18	0.78	0.89	0.78	0.78	0.88	1.01	0.91	1.04

The CS and ASS evaluation results of 3 kinds of pediatric compound amino acid injection are shown in Table 6.

**Table 6:** The CS and ASS evaluation results of 3 varieties of pediatric compound amino acid injections.

	18AA-I		18AA-2		19AA-1	
	CS	AAS	CS	AAS	CS	AAS
Lysine	1.74	1.61	1.43	1.32	1.08	0.99
Valine	0.84	0.80	1.03	0.99	1.09	1.05
Threonine	1.18	1.01	0.77	0.66	0.82	0.70
Isoleucine	0.88	0.87	1.31	1.28	1.40	1.37
Tryptophan	1.27	1.56	1.02	1.26	1.08	1.34
Methionine + cysteine	0.62	0.73	0.59	0.66	0.59	0.70
Leucine	1.25	1.12	1.41	1.26	1.50	1.34
Phenylalanine + tyrosine	0.55	0.61	0.54	0.60	1.08	0.99

The results in Tables 4 and 5 showed that compared with whole egg protein, the first limiting amino acid of HA was lysine, with a value of 0.15. Among the five varieties of adult CBAAI, 18AA-V had the lowest value (0.59), and 18AA-II had the highest value (0.78) [8]. Compared with the FAO/WHO standard protein, the first limiting amino acid of HA was lysine, with a value of 0.19 (*P*<0.05). Among the five varieties of adult balanced compound amino acid injections, 18AA-V had the lowest value (0.58), and 18AA-II had the highest value (0.74) [7]. The first limiting amino acid of HA had far lower nutritional value than 18AA-V.

The results in Tables 4 and 6 showed that compared with whole egg protein, the value of the first limiting amino acid of HA, 18AA-I_p_, 18AA-II_p_, and 19AA-I_p_ were 0.15, 0.55, 0.54, and 0.59, respectively (*P*<0.05). Therefore, the first limiting amino acid of HA had far lower nutritional value than 18AA-I_p_, 18AA-II_p_, and 19AA-I_p_.

The utility of HA and 5 adult CBAAIs in HA synthesis was evaluated using EAAI, SRCAA, and their similarity to egg protein and FAO/WHO models. The results indicated that HA had an EAAI value of 78.23, suggesting that its comprehensive quality of EAA is inferior when compared to egg protein. The SRCAA value for HA was 46.50, which implies a limited value in the synthesis of HA. Furthermore, the closeness of HA to egg protein was 0.81, and to the FAO/WHO model, it was 0.76, both of which do not meet the ideal criteria. This indicates a significant disparity between the amino acid composition of HA and the standard protein patterns. The EAAI, SRCAA, and closeness evaluation results of HA, 5 varieties of amino acids for injection, and 3 varieties of pediatric CBAAI are shown in Table 7.

**Table 7:** EAAI, SRCAA and closeness evaluation results.

	18AA	18AA-I	18AA-II_original research_	18AA-II (8.5%)	18AA- IV	18AA-V	18AA-II_p_	18AA-I_p_	19AA-I_p_
EAAI	97.7	97.1	98.55	98.55	93.21	92.15	98.09	99.62	101.49
SRCAA	84.76	82.76	76.69	76.69	75.69	77.72	39.90	41.00	41.57
Egg closeness	0.94	0.94	0.94	0.94	0.92	0.91	1.18	1.00	1.18
FAO/WHO closeness	0.85	0.91	0.90	0.90	0.85	0.87	1.54	1.36	1.54

The results in Table 7 showed that the values of EAAI, SRCAA, the Egg closeness, and the FAO/WHO closeness of 18AA-Ⅴ and 18AA-II were (92.15, 77.72, 0.91, 0.87) and (98.55, 76.69, 0.94, 0.90), respectively. Therefore, the EAA comprehensive evaluation of five CBAAI was higher than that of HA. The values of EAAI, SRCAA, the Egg closeness, and the FAO/WHO closeness of 18AA-I_p_, 18AA-II_p_, and 19AA-I_p_ were (99.62, 41.00, 1.00, 1.36), (98.09, 39.90, 1.18, 1.54), and (101.49, 41.57, 1.18, 1.54), respectively. The values of EAAI, the Egg closeness, and the FAO/WHO closeness of three varieties of paediatric CBAAI were higher than that of HA, and only the SRCAA was slightly lower than that of HA. The comparison of CS and AAS of HA and different amino acid injections is shown in Table 8.

**Table 8:** Comparison of CS and AAS between HA and different amino acid injections.

Amino acid injection type	HA	18AA-II	3AA	9AA	18AA-VII
CS	0.81±0.05	0.94±0.04	0.58±0.03	0.78±0.04	0.91±0.05
AAS	0.76±0.04	0.90±0.03	0.59±0.02	0.77±0.03	0.87±0.04
T value	\	4.23	6.15	3.87	5.56
*P* valve	\	<0.001	<0.001	<0.001	<0.001

The statistical analysis results showed that compared with HA, 18AA-II, 3AA, 9AA, and 18AA-VII had significant differences in CS and AAS (*P*<0.05). These results indicated that 18AA-II, 3AA, 9AA, and 18AA-VII were more valuable than HA in the synthesis of HA.

### 4.2 Evaluation results of the value of HA and therapeutic amino acid injections in synthesizing HA

Compound amino acid 3AA injection contains three kinds of BCAA: valine, isoleucine and leucine. The content of BCAA is 100%. The percentage of BCAA and AAA in HA are 19.15% 80.85%, respectively. The amino acid spectrum of compound amino acid 3AA injection is more consistent with that of patients with liver injury. Therefore, compound amino acid 3AA injection is superior to HA in treating hypoalbuminemia with liver injury.

Compound amino acid 9AA injection contains 8 kinds of EAA and histidine (semi EAA). The percentage of EAA and NEAA in HA are 48.25% and 51.75%, respectively. Therefore, the amino acid composition of 9AA is more consistent with the plasma amino acid profile of patients with acute and chronic renal insufficiency and severe renal failure. Therefore, compound amino acid 9AA injection is superior to HA in treating hypoalbuminemia with renal injury.

The percentage of BCAA content in 18AA-Ⅶ is 36.5%, while that in HA is only 19.15%. The content of BCAA in HA is too low. The amino acid composition of 18AA-VII is more consistent with the plasma amino acid profile of trauma patients. Further statistical analysis shows that 3AA injection is significantly more valuable than HA in synthesizing HA in patients with abnormal liver function (*P*<0.01). Similarly, 9AA injection shows significantly higher value compared to HA in patients with renal dysfunction (*P*<0.01). The value of 18AA-VII injection in the synthesis of HA is also significantly higher than that of HA in trauma patients (*P*<0.01). Therefore, compound amino acid 18AA-VII injection is superior to HA in treating hypoalbuminemia with trauma.

## 5. Discussion

For evaluating a drug, safety, efficacy, and economy should be considered at the same time. Regarding safety, raw material for HA comes from human blood. Although the raw plasma is screened for related pathogens, and the measures of removing and inactivating viruses are added during the production process, there is still a potential risk of transmitting some known and unknown pathogens. The amino acid injection is a sterile aqueous solution comprising various amino acids. The incidence and severity of adverse reactions of HA is much higher than that of amino acid injection.

Regarding effectiveness, if the human body needs to synthesize albumin, it needs to simultaneously receive enough energy from drugs such as fat emulsion and glucose. As long as there is enough energy support, the balanced amino acid injection can provide essential and non-EAA to synthesize HA. Research has shown that among the five adult CBAAIs, even 18AA-V and EAA, which have the lowest first limiting amino acid index, have a much higher overall quality than HA. In addition, the half-life of HA is about 12.7–18.2 days [16], and it takes time to decompose into free amino acids, which is not possible to quickly synthesize HA after entering the human body like a balanced amino acid injection. Therefore, for adult patients, even if sufficient energy support is provided, the effect of HA on hypoalbuminemia is extremely poor, and the CBAAI should be the first choice [17].

Taurine is added to three kinds of paediatric CBAAI (18AA-I_p_, 18AA-II_p_, and 19AA-I_p_), which can protect cell membrane, promote brain development, and maintain normal retinal function. Combined with bile, it can facilitate bile acid metabolism, prevent cholestasis, enhance myocardial cell function, and help children’s growth and development. In addition, the liver enzyme system of infants is not mature, and the metabolism of amino acids in infants is different from that in adults. Tyrosine, cysteine, histidine, and taurine are added to the formula, while phenylalanine, methionine, and glycine are reduced to better meet the nutritional needs of children. The research results show that the first limiting amino acid of the three kinds of paediatric CBAAI is much higher than HA, and the comprehensive quality of EAA is also better than that of HA. Therefore, paediatric CBAAI should be the first choice for children with hypoproteinemia, which is more conducive to children’s albumin synthesis. However, the amino acid spectrum of HA does not align with the content of taurine, cysteine, and phenylalanine, which are present in low and high quantities, respectively. Therefore, HA has a poor effect in synthesizing children’s HA.

The content of BCAA in compound amino acid 3AA injection is 100%, which conforms to the amino acid spectrum of patients with liver injury. When treating hypoalbuminemia with liver injury, 3AA is the first choice, which can not only prevent and treat hepatic encephalopathy, but also synthesize HA well. The catabolic products of HA contain a large number of AAA, including 19.15% BCAA and 80.85% AAA, which are inconsistent with the amino acid profile of patients with liver injury. When HA is used to treat hypoalbuminemia with moderate to severe liver dysfunction, it is not only ineffective in synthesizing HA, but also may further damage liver function, and even cause hepatic encephalopathy.

The amino acid composition of compound amino acid 9AA injection matches the plasma amino acid profile of patients with renal injury. When treating hypoalbuminemia with kidney injury, compound amino acid 9AA injection should be the first choice, which can well synthesize HA and reduce azotemia. However, the catabolic products of HA in vivo contain a lot of NEAA, including 48.25% of EAA and 51.75% of NEAA, which do not match the changes of plasma amino acid profile of patients with renal function injury. When treating hypoalbuminemia with moderate to severe renal dysfunction, HA cannot well synthesize HA, which may further damage renal function.

Regarding the economy, the nitrogen content of protein is about 16%. 1g of nitrogen is equivalent to 6.25g of amino acids, and it is generally believed that 1g of protein is equivalent to 1g of amino acids. The amount of albumin required by different patients every day is about 0.6–3 g/(kg·d). When parenteral nutrition support is required for patients who cannot eat or who do not eat enough, the amino acid requirement is 0.6–3 g/(kg·d). In China, 10g amino acid only requires 5–10 yuan (RMB), while 10g HA requires 400–500 yuan (RMB). To achieve the same effect of amino acids and HA, the latter is extremely expensive.

The American Hospital Association Guidelines for the Use of HA, Non-Protein Colloids and Crystalloid Solutions and the European Recommendations for the use of Immune Globulin and Albumin both suggest that HA should not be used in patients requiring nutritional intervention [18,19]. Malnutrition is only one of the causes of hypoalbuminemia. Many countries still use white protein for treatment of hypoalbuminemia caused by other reasons, such as liver function damage, liver dysfunction, trauma, long-term protein loss (such as gastrointestinal ulcers, hemorrhoids, menstrual disorders, nephrotic syndrome, etc.), cancer cachexia, severe infections, etc. The use of HA in China is more unreasonable. The drug instructions for HA in China also indicate that HA can be utilized as a nutritional supplement in pharmacological contexts, particularly when nitrogen metabolism is impaired. In such instances, HA can be employed as a nitrogen source to provide nutrition for tissues. Due to the vague instructions, about 33% of HA is sold as nutritional supplement for patients with nutritional risks and malnutrition in China’s township health centers, county-level hospitals, municipal-level hospitals, and provincial-level hospitals [20]. According to statistics, in China, a total of 59.91 million bottles of HAS are issued in 2020, with an increase of 19.04% year-on-year [21]. If calculated at the proportion of 33%, in 2020, the consumption of HSA for nutritional treatment alone in China is at least 80 million RMB. Based on the above discussion and analysis, many countries in the world are using unreasonably and wasting HA resources.

## 6. Conclusion

This study suggested that the safety, effectiveness, and economy of CBAAI and therapeutic compound amino acid injections are far superior to HA in the treatment of hypoproteinemia. Amino acid injections are preferred to HA in the treatment of hypoproteinemia. Therefore, countries all over the world urgently need to formulate relevant specifications for the rational use of HA, modify the imperfections of albumin drug instructions, and reduce resource waste.
